# Technological innovations and breakthrough pathways in syphilis diagnosis: addressing global resurgence and the journey toward elimination

**DOI:** 10.3389/fcimb.2026.1781460

**Published:** 2026-04-01

**Authors:** Juan Hu, Chunyan Tan, Qin Li, Yanting Liu, Guangjun Xiao

**Affiliations:** Department of Clinical Laboratory, Suining Central Hospital, Suining, Sichuan, China

**Keywords:** diagnostic algorithms, molecular diagnostics, point-of-care testing, syphilis, *Treponema pallidum*

## Abstract

The global resurgence of syphilis, characterized by a precipitous rise in incidence and the persistence of congenital transmission, has necessitated a critical re-evaluation of traditional diagnostic paradigms. This review synthesizes recent technological innovations in the detection of *Treponema pallidum*, spanning from molecular precision to decentralized point-of-care (POC) accessibility. This review synthesizes the paradigm shift from traditional darkfield microscopy (DFM) toward advanced nucleic acid amplification tests (NAATs). By evaluating innovations such as CRISPR-based assays and transcription-mediated amplification (TMA), we highlight their capacity to bridge the diagnostic ‘window period’ where traditional serology often fails. Furthermore, the review evaluates the clinical adaptability of automated reverse-sequence algorithms and the utilization of signal-to-cutoff (S/Co) ratios to optimize high-throughput laboratory workflows. Significant attention is given to breakthroughs in biosensing—such as silk cocoon membrane-integrated platforms—and the role of novel biomarkers like IgA and proteomic arrays in differentiating active infection from historical exposure. The significance of this review lies in its comprehensive mapping of “breakthrough pathways” that address diagnostic bottlenecks in complex manifestations like neurosyphilis and neonatal cases. Evidence suggests that while these technological breakthroughs possess transformative potential, their global scalability remains constrained by a dual challenge: the lack of standardized commercial platforms and the diagnostic ambiguity posed by the ‘serofast’ state. Consequently, the integration of these innovations into routine practice requires moving beyond isolated tools toward a cohesive, multimodal diagnostic framework Future perspectives emphasize the integration of smartphone-based AI diagnostics and the necessity of aligning technological advancements with biomedical prevention strategies, such as DoxyPEP and multi-epitope vaccine development. Ultimately, establishing a multi-modal diagnostic ecosystem that prioritizes health equity and real-time surveillance is essential for dismantling the systemic barriers to global syphilis elimination.

## 1 Introduction

Treponema pallidum, the causative agent of syphilis, has transitioned from a once-marginalized historical pathogen to a formidable contemporary epidemic (Hook, 2017; Gottlieb et al., 2024; WHO, 2024; Rosset et al., 2025). Recent global surveillance indicates a precipitous resurgence, with new cases among adults rising from 7.1 million in 2020 to 8 million in 2022 (New report flags major increase in sexually transmitted infections, amidst challenges in HIV and hepatitis; GBD 2021 Sexually Transmitted Infections Collaborators, 2024). This alarming trend is mirrored in regional data across Japan, Canada, and the United States, where congenital syphilis cases have seen an 11-fold increase since 2005 (Kimball et al., 2020; John et al., 2024; Komori et al., 2024; Gregory and Ely, 2024; Azqul et al., 2025; Mackrell et al., 2025). The burden is disproportionately concentrated among vulnerable groups, particularly men who have sex with men (MSM), where global pooled prevalence has reached 10.4% (Zheng et al., 2024). Despite its curability, syphilis continues to impose the highest age-standardized disability-adjusted life-year (DALY) rate of any bacterial infection globally, a crisis exacerbated by shifting sexual behaviors and COVID-19 related healthcare disruptions (Chen et al., 2024; Rosset et al., 2025). This situation is fundamentally complicated by the pathogen’s “stealth” nature and its ability to function as the “great imitator,” presenting with diverse manifestations ranging from primary lesions to severe neurological complications (Zhou et al., 2024).

Traditional diagnostic paradigms, while foundational, are frequently compromised by inherent technical limitations. Nontreponemal assays are often prone to biological false-positives in specific clinical contexts or false-negatives during the early primary stage (Simpore et al., 2022; Treger et al., 2025), while treponemal-specific tests generally remain reactive for life, failing to distinguish active infection from treated historical cases (John et al., 2024). Specialized manifestations like neurosyphilis present further hurdles due to the low specificity of cerebrospinal fluid tests resulting from antibody diffusion (Alberto et al., 2024). To bridge these gaps, a robust movement toward technological innovation has emerged, driven by the decentralization of testing through point-of-care (POC) accessibility to enable “test-and-treat” strategies (Chitneni et al., 2023; Mackrell et al., 2025). Simultaneously, the pursuit of molecular precision has yielded next-generation nucleic acid amplification tests (NAATs), such as CRISPR-LwCas13a assays, which achieve single-copy sensitivity and offer potential for real-time surveillance (Chen et al., 2022).

**Figure 1:**
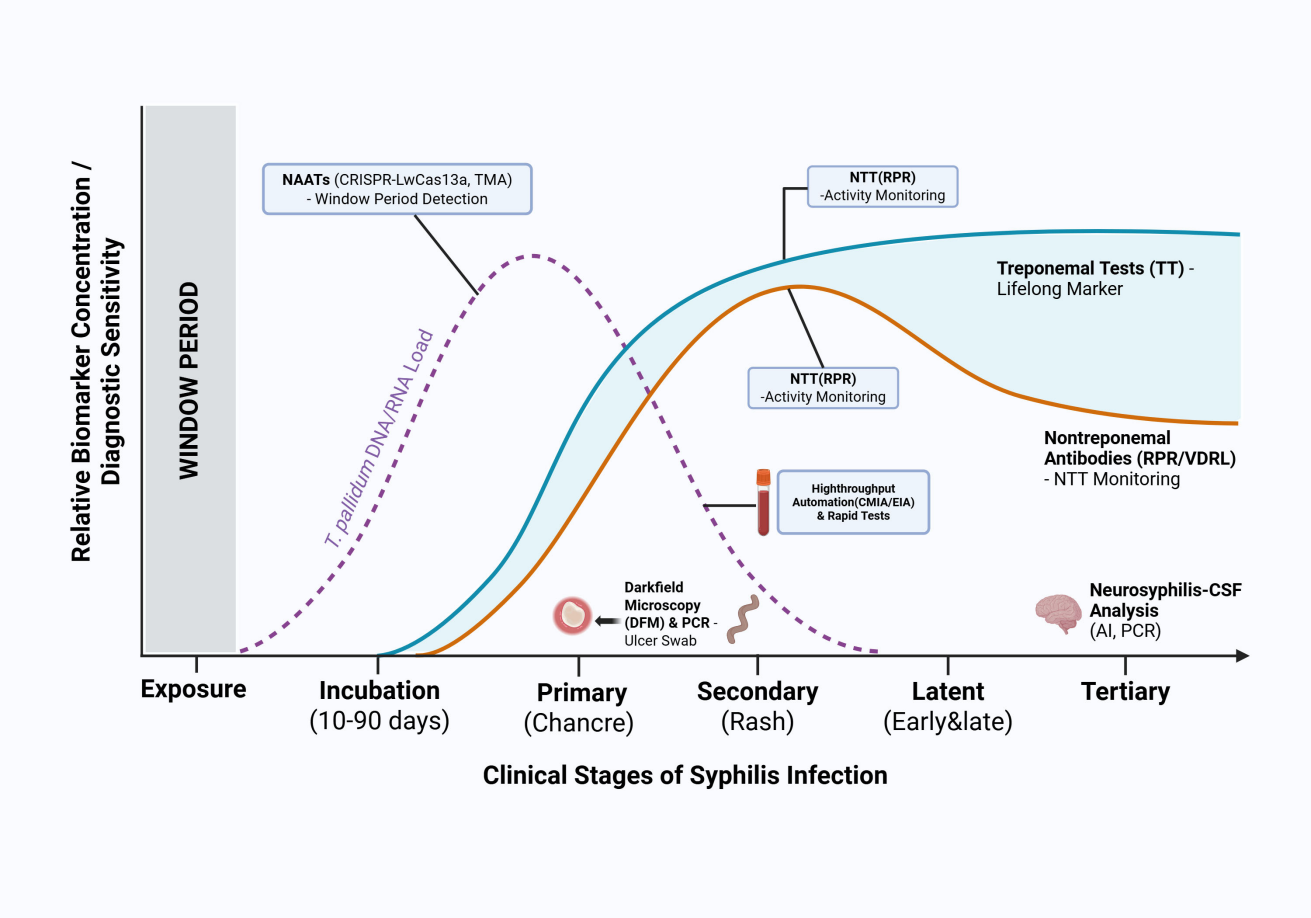
Temporal dynamics of pathogen load and serological biomarkers throughout the clinical progression of syphilis. This figure delineates the diagnostic utility and relative concentration of various biomarkers across the incubation, primary, secondary, latent, and tertiary stages of infection. The initial window period following exposure is marked by the ascent of *T. pallidum* DNA/RNA, where advanced nucleic acid amplification tests (NAATs)—including CRISPR-LwCas13a and transcription-mediated amplification (TMA)—provide significant sensitivity. Direct detection methods, such as DFM and PCR, are most efficacious during the primary stage when lesions (chancres) are present. Upon seroconversion, the transition to secondary and latent syphilis is characterized by the sustained reactivity of treponemal-specific tests (TT), serving as lifelong markers, and the fluctuating titers of nontreponemal tests (NTT), which are essential for longitudinal monitoring of treatment response. The model further integrates specialized diagnostic pathways, such as the Antibody Index (AI) for identifying neurosyphilis and automated high-throughput platforms for efficient laboratory workflows.

To address large-scale screening requirements, high-throughput automation and signal-to-cutoff (S/Co) optimization have become cornerstones of modern laboratory medicine (Demir Çuha et al., 2022; Cheng et al., 2024). Furthermore, the identification of novel biomarkers—including *T. pallidum*-specific IgA responses and minimal proteomic arrays—offers improved capacity to differentiate active infection from historical exposure (Haynes et al., 2023; Rodríguez et al., 2023). Supported by digital bioanalysis and cloud-based data synchronization, these innovative diagnostic pathways facilitate more efficient screening and clinical management, addressing existing operational obstacles to infection control (He and Xiao, 2025; Wall et al., 2025; Yaping et al., 2025). This review synthesizes these recent technological innovations, evaluating their clinical adaptability and mapping their integration into a multimodal diagnostic ecosystem essential for global syphilis elimination. The temporal dynamics of *T. pallidum* load and the corresponding emergence of various diagnostic biomarkers across different clinical stages of infection are illustrated in **Figure 1**. As demonstrated by these trajectories, the initial “window period” following exposure represents a critical diagnostic blind spot; during this phase, the pathogen load may reach high levels while host antibodies remain undetectable, rendering traditional serological assays inherently ineffective (Hughes et al., 2025). This biological lag underscores the necessity of integrating molecular precision, such as NAATs, to achieve early detection during the pre-seroconversion phase (Sweitzer et al., 2025). Furthermore, the fluctuating dynamics between pathogen dissemination and host immune response across clinical stages reveal that no single diagnostic modality is sufficient for all manifestations, necessitating a cohesive, multimodal framework to bridge existing gaps in infection control (John et al., 2024).

## 2 Methodology

To ensure a rigorous synthesis of the diagnostic landscape for syphilis, a systematic literature search was conducted across PubMed, Web of Science, and Scopus databases. The literature search primarily focused on the period from 2010 to 2025 to capture recent technological innovations. Additionally, foundational historical literature (dating back to 1946) was selectively included to provide essential context on the evolution of diagnostic standards and benchmarks. A specific emphasis was placed on high-impact studies and clinical guidelines published between 2024 and 2025.

Search strings utilized Boolean operators to combine terms including: ‘Treponema pallidum diagnostic algorithms’, ‘reverse sequence algorithm (RSA)’, ‘signal-to-cutoff (S/Co) ratio’, ‘CRISPR-based detection’, ‘transcription-mediated amplification (TMA)’, and ‘point-of-care testing (POCT)’. Additionally, keywords such as ‘diagnostic window’ and ‘molecular-serological complementarity’ were included to address the integration of NAATs with traditional serology.

## 3 Serological assays and diagnostic algorithms

### 3.1 Traditional vs. nontreponemal tests

Modern syphilis diagnostics remain predicated upon a dual-test serological framework, which bifurcates into nontreponemal tests (NTT) and treponemal tests (TT). NTTs, such as the Venereal Disease Research Laboratory (VDRL) and Rapid Plasma Reagin (RPR), identify IgG and IgM antibodies directed against lipoidal antigens (cardiolipin, lecithin, and cholesterol) released following host cellular damage (Harris et al., 1946; Haynes et al., 2023; John et al., 2024). While NTTs are indispensable for monitoring treatment response via titer quantification, they are susceptible to false-positive reactions in patients with atopy, pregnancy, or other systemic conditions (Jurado, 1996; John et al., 2024; Treger et al., 2025) Conversely, TTs target specific *T. pallidum* proteins (e.g., Tp0435 and Tp0574), providing high specificity as confirmatory markers (Haynes et al., 2023; John et al., 2024). Traditional modalities like TPPA are increasingly being superseded by high-throughput automated platforms (ELISA/CMIA), which form the foundation of modern algorithms (Silva et al., 2024; Saldarriaga et al., 2025).

### 3.2 The shift to Reverse Sequence Algorithm

A fundamental paradigm shift in syphilis screening is the transition from the Traditional Algorithm (TA)—which initiates with an NTT—to the Reverse Sequence Algorithm (RSA), prioritizing automated TTs as the primary screening tool. This transition is driven by the need for high-throughput capacity and the superior sensitivity of RSA in identifying late latent syphilis cases that are frequently overlooked by primary RPR screening (Morshed, 2014; Morshed et al., 2022).

In an RSA workflow, specimens are initially screened via automated TTs; reactive specimens then undergo reflex testing with an NTT to distinguish between active infection and “immunological scars” from successfully treated historical cases (Morshed et al., 2022). While the TA remains cost-efficient in low-prevalence settings, its manual labor intensity and vulnerability to missing very early or late latent cases have made the RSA a more adaptable choice for large-scale clinical laboratories (Saldarriaga et al., 2025; Sweitzer et al., 2025). The adoption of RSA is consistently supported by international consensus, including European (IUSTI) and UK guidelines, which emphasize its high-throughput advantages (Kingston et al., 2016; Janier et al., 2021).

### 3.3 Signal-to-Cutoff ratio optimization

In the implementation of the RSA, the **Signal-to-Cutoff (S/Co)** ratio has emerged as a critical metric for enhancing diagnostic specificity and optimizing laboratory workflows. While automated TTs offer high efficiency, their low positive predictive value (PPV) in low-prevalence populations often leads to false-positive results, necessitating rigorous confirmation via TPPA (Zulu et al., 2024).

**Figure 2:**
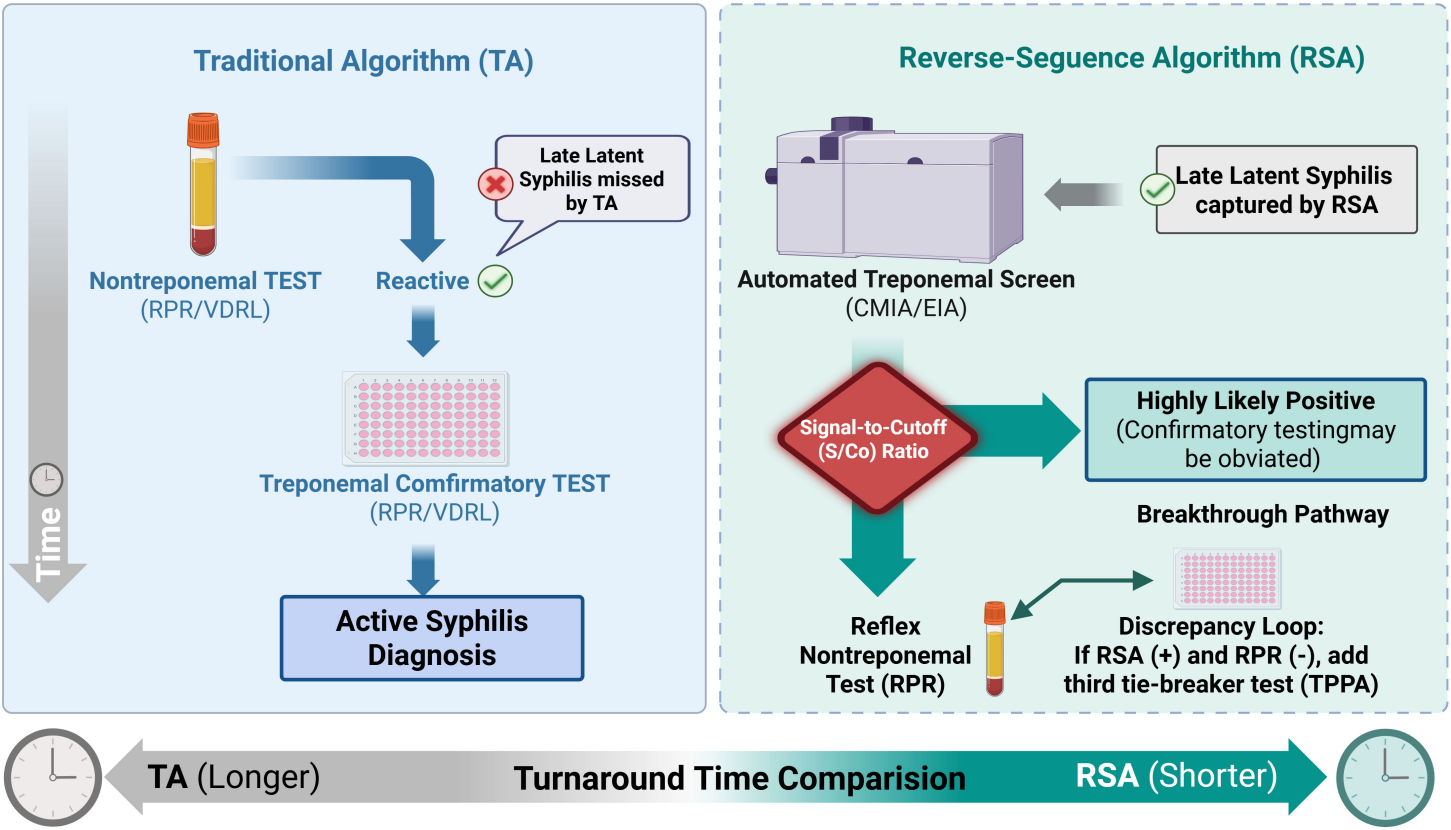
Comparative workflows of traditional versus reverse-sequence syphilis screening algorithms. This diagram illustrates the divergent diagnostic pathways dictated by the initial screening methodology. The Traditional Algorithm (TA), depicted on the left, initiates with a manual nontreponemal test (NTT), requiring treponemal text (TT) confirmation for reactive results; this approach is highly specific but may lack sensitivity in very early or late latent stages. The Reverse-Sequence Algorithm (RSA), shown on the right, is driven by high-throughput automated TT immunoassays as the primary screen, enhancing the detection of latent infections. A critical optimization pathway within the RSA is the integration of the quantitative Signal-to-Cutoff (S/Co) ratio as a decision node; an S/Co value ≥ 10.4 is associated with near 100% specificity, allowing for the bypass of secondary confirmation, whereas lower ratios require reflex NTT testing to resolve potential discordant results (TT-reactive/NTT-nonreactive).

Current clinical evidence suggests that optimizing S/Co thresholds can effectively mitigate the need for redundant confirmatory testing. High S/Co values (e.g., ≥ 10.4 in specific automated assays) have been shown to achieve near 100% specificity, potentially bypassing the requirement for NTT or TPPA verification in certain contexts. Conversely, lower S/Co values (e.g., between 1.0 and 5.0) are frequently associated with false-positives or resolved infections, requiring mandatory supplemental testing (Workowski et al., 2021; Park et al., 2023; Cheng et al., 2024). Integrating these optimized thresholds not only reduces turnaround time (TAT) but also provides a cost-effective strategy by focusing resources on samples with ambiguous serological profiles. Furthermore, the implementation of optimized S/Co thresholds is essential in high-throughput RSA workflows to mitigate the confirmatory testing burden caused by the high analytical sensitivity of automated treponemal assays, which can otherwise lead to excessive false-positive results in low-prevalence populations (Cheng et al., 2024). A comparative overview of the traditional screening algorithm and the reverse sequence algorithm (RSA), emphasizing the critical decision nodes and the role of S/Co ratio optimization, is presented in **Figure 2**.

### 3.4 Clinical and economic evaluation

The clinical adaptability of these algorithms involves a complex trade-off between sensitivity and resource allocation. From a public health perspective, the RSA’s ability to enhance case-finding is significant; for instance, implementation in British Columbia identified over 4,000 latent cases that traditional screening would have missed (Morshed et al., 2022). However, the RSA may lead to a three-fold increase in overtreatment rates in low-prevalence cohorts, such as maternal health screenings, due to the detection of non-active infections (Zulu et al., 2024). The diagnostic performance and clinical utility of emerging serological biomarkers, including specific IgA responses and novel membrane proteins, are summarized in **Table 1**.

**Table 1:** Performance metrics of novel serological markers and diagnostic assays for syphilis.

Category	Assay/marker	Target/stage	Sensitivity	Specificity	Key utility	References
Immunoglobulin	Anti-T. pallidum IgA	Primary/Secondary/Latent	80.3%–100%	98.10%	Differentiating active from past infection	(Rodríguez et al., 2023)
Recombinant Protein	TpN17 (Indirect ELISA)	Primary/Secondary/Latent	93.2%–100%	100%	High-accuracy stage-specific screening	(Silva et al., 2024)
Decentralized Testing	Hightop Syphilis RDT	Symptomatic/Asymptomatic	97.5%–100%	98.5%	Rapid triage in resource-limited areas	(Ngounouh et al., 2025)
Semi-Quantitative	LISA (TP15, TP17, TP47)	Overall diagnosis	Up to 98.8%	99%	Therapeutic monitoring	(Ke et al., 2024)
Specialized	Intrathecal IgG Index	Neurosyphilis/CNS involvement	90.70%	100%	Confirmatory tool for neurosyphilis	(Alberto et al., 2024)

AI, antibody index; CLIA, chemiluminescence immunoassay; EIA, enzyme immunoassay; FTA-ABS, fluorescent treponemal antibody absorption; NTT, nontreponemal test; POCT, point-of-care testing; RPR, rapid plasma reagin; RSA, reverse sequence algorithm; S/Co, signal-to-cutoff; TA, traditional algorithm; TPPA, Treponema pallidum particle agglutination; TT, treponemal test; VDRL, Venereal Disease Research Laboratory.

Economic evaluations suggest that while the RSA is a vital intervention for preventing adverse outcomes like congenital syphilis, it yields a high incremental cost-effectiveness ratio (ICER) unless the local prevalence exceeds a 6% threshold (Megha et al., 2024; Saldarriaga et al., 2025). Despite these economic complexities, the transition to RSA, supported by S/Co optimization and automated platforms, represents a “breakthrough pathway” in modern diagnostic management, streamlining clinical pathways and ensuring more robust screening coverage, particularly in high-risk populations and prenatal care (Timothy et al., 2024; Saldarriaga et al., 2025).

## 4 Molecular diagnostics: from PCR to next-generation tools

### 4.1 NAATs and the resolution of the diagnostic ‘window period’

The diagnostic landscape is currently undergoing a transformative transition from manual visualization toward sophisticated molecular innovations (Hay et al., 1990; Sweitzer et al., 2025). As illustrated in **Figure 1**, traditional serology is frequently compromised by an inherent “window period” following exposure, during which *Treponema pallidum* (TP) disseminates systemically while host antibodies remain below detectable thresholds (Treger et al., 2025). To bridge this gap, Nucleic Acid Amplification Tests (NAATs), specifically those leveraging Polymerase Chain Reaction (PCR), have emerged as superior alternatives due to their high specificity and capacity to detect TP across diverse specimens—ranging from primary lesions to paucicellular late-stage samples (Sweitzer et al., 2025). Meta-analytical data underscores this performance, with pooled sensitivities for conventional and real-time PCR reaching approximately 77.52% and 68.43%, respectively, while maintaining specificities above 98% (Simpore et al., 2022).

Beyond conventional DNA-based PCR, Transcription-Mediated Amplification (TMA) has demonstrated significant clinical utility by targeting 16S ribosomal RNA (rRNA) (Zondag et al., 2023). The TP-TMA assay effectively increases case-finding rates by identifying infections missed by routine serology, particularly during the incubation phase (Zondag et al., 2023). Furthermore, TMA’s isothermal nature and high analytical sensitivity in rectal swabs highlight its potential for enhanced epidemiological surveillance in high-risk cohorts without cross-reactivity from commensal species (Lutz et al., 2023; Krueger et al., 2025).

### 4.2 CRISPR-cas systems: the next frontier of ultrasensitive detection

The pursuit of molecular precision has yielded next-generation systems such as CRISPR-LwCas13a assays, which achieve single-copy sensitivity and offer significant potential for real-time surveillance (Chen et al., 2022). The technical superiority of this platform lies in its “collateral cleavage” mechanism: upon the crRNA-guided recognition of a specific TP RNA sequence, the Cas13a protein undergoes a conformational change that activates its non-specific RNase activity. This triggered enzyme then indiscriminately cleaves nearby fluorescently-quenched RNA reporters, resulting in an exponentially amplified signal that achieves a much lower limit of detection (LOD) than conventional methods (Chen et al., 2022). By transitioning from standard target genes to these innovative CRISPR-based platforms, the diagnostic framework can effectively address paucicellular manifestations where traditional PCR might fail (Chen et al., 2022; Lu et al., 2024).

### 4.3 Innovative biomarkers for infection staging and the ‘serofast’ challenge

To address the diagnostic ambiguity posed by the ‘serofast’ state, recent molecular studies have identified novel biomarkers designed to differentiate active metabolic activity from historical exposure (Rodríguez et al., 2023).

Specific Membrane Proteins: The protein Tp0136 has been shown to induce spheroidization of vascular endothelial cells, thereby widening intercellular junctions and enhancing vascular permeability, serving as a promising biomarker for early-stage detection (Lin et al., 2025).

Anti-TP IgA Response: The anti-*T. pallidum* IgA response has emerged as a transient marker that correlates more closely with active infection than lifelong IgG markers, offering a potential solution to the common diagnostic challenge in treated historical cases (Rodríguez et al., 2023).

Semi-Quantitative Analysis: The utilization of semi-quantitative luciferase immunosorbent assays (LISA) targeting antigens like TP15, TP17, and TP47 provides enhanced clinical decision support for treatment monitoring and overall diagnosis (Ke et al., 2024).

### 4.4 Current limitations and obstacles to clinical implementation

While emerging molecular tools offer high sensitivity, several systemic barriers hinder their widespread clinical adoption (Sweitzer et al., 2025). The high cost of specialized reagents and the requirement for sophisticated equipment, such as fluorescence readers, remain prohibitive for base-level hospitals (Ahmadi et al., 2025). Economic evaluations suggest that while these advanced algorithms are vital for preventing adverse outcomes, they may yield a high incremental cost-effectiveness ratio (ICER) unless the local prevalence exceeds specific thresholds, such as 6% (Megha et al., 2024). Furthermore, the lack of standardized external quality assessment (EQA) programs for novel biomarkers like Tp0136 or IgA makes it difficult to guarantee inter-laboratory reproducibility (Hughes et al., 2025) (**Table 2**).

**Table 2:** Comparative performance metrics and clinical utility of novel molecular and serological syphilis diagnostic assays.

Diagnostic category	Specific assay/marker	Target specimen(s)	Reported sensitivity (%)	Reported specificity (%)	Key clinical advantage/limitation	References
Molecular Diagnostics	PCR (targeting polA, tpp47, etc.)	Lesion exudates, Urine, Tissue	~70% – 80%	> 98%	Advantage: Early diagnosis in seronegative window period. Limitation: Cannot distinguish viable from dead organisms.	(Simpore et al., 2022)
Next-Gen Molecular	**CRISPR-LwCas13a Platforms**	Clinical isolates/samples	Near single-copy detection limits	High (Sequence-specific)	Advantage: Next-generation ultrasensitive technology; potential for rapid POC.	(Chen et al., 2022)
Novel Serological Markers	Anti-T.p. IgA	Serum/Plasma	80.3% – 100%	~ 98.1%	Advantage: Potential to distinguish active infection from past (treated) infection.	(Rodríguez et al., 2023)
Specialized Diagnostics	**CSF-Antibody Index (AI)**	Paired CSF & Serum	~ 90.7%	up to 100%	Advantage: Critical adjunct supplement to the gold standard for neurosyphilis diagnosis.	(Alberto et al., 2024)
Advanced Serology	Line Immunoassay (LISA) (recombinant antigens TP15/17/47)	Serum/Plasma	~ 98.8%	~ 99.0%	Advantage: Useful for confirming discrepant results; potential for semi-quantitative analysis or staging.	(Ke et al., 2024)

AI, antibody index; AMR, antimicrobial resistance; CSF, cerebrospinal fluid; CRISPR, clustered regularly interspaced short palindromic repeats; LISA, line immunoassay; LOD, limit of detection; mNGS, metagenomic next-generation sequencing; NAATs, nucleic acid amplification tests; PCR, polymerase chain reaction; S/Co, signal-to-cutoff; TMA, transcription-mediated amplification; TP-IgA, Treponema pallidum-specific immunoglobulin A.

Bold values indicate superior sensitivity or specificity metrics within specialized diagnostic scenarios.

## 5 Specialized diagnostic scenarios

### 5.1 Neurosyphilis: overcoming the limitations of direct and standard detection

The diagnosis of neurosyphilis (NS) remains a significant clinical challenge due to the lack of a definitive “gold standard” and the “stealth” nature of *T. pallidum* in the central nervous system (CNS) (Alberto et al., 2024). Traditional direct detection methods, such as darkfield microscopy (DFM), are inherently inapplicable to cerebrospinal fluid (CSF) samples, while the low spirochete load in late-stage CNS involvement often yields false-negative results in conventional PCR assays (Zhou et al., 2022).

Historically, the CSF-VDRL test has served as the laboratory cornerstone; however, while highly specific, its clinical utility is constrained by a sensitivity as low as 73%–83% (Xie et al., 2023). Conversely, treponemal assays (TPPA/EIA) offer superior sensitivity but fail to distinguish between antibodies endogenously produced within the CNS and those resulting from passive diffusion across a compromised blood-brain barrier (BBB) (Alberto et al., 2024).

To resolve this ambiguity, the Antibody Index (AI) represents a technical breakthrough by quantifying the intrathecal synthesis of specific IgG antibodies (Alberto et al., 2024). By calculating the ratio of CSF/serum specific antibodies relative to the total IgG or albumin ratio, the AI effectively accounts for BBB integrity. An AI threshold of ≥1.7 has demonstrated a specificity of up to 100%, providing a robust confirmatory tool even when conventional serology is discrepant (Zhou et al., 2022). Furthermore, metagenomic next-generation sequencing (mNGS) has emerged as a potent supplementary tool, capable of detecting “stealth” TP DNA in symptomatic patients with normal MRI findings, thereby closing the gap left by traditional paucicellular detection limits (Zhou et al., 2022).

### 5.2 Decentralized Point-of-Care Testing and biosensing innovations

The global resurgence of syphilis has catalyzed a shift toward decentralized diagnostics to reduce the diagnostic-to-treatment interval (Pham et al., 2022). Modern Point-of-Care Testing (POCT) platforms have evolved beyond simple screening to address complex clinical needs:

Addressing Access and Stigma: The First to Know (FTK) Syphilis Test is the first FDA-authorized over-the-counter (OTC) treponemal assay for self-testing (Clark et al., 2025). With 93.4% sensitivity and 99.5% specificity, it bypasses systemic barriers such as clinical stigma, facilitating earlier entry into the care continuum (Clark et al., 2025).

Differentiating Active Infection: The DPP Syphilis Screen & Confirm Assay utilizes dual-path technology to simultaneously detect treponemal and non-treponemal antibodies from a single fingerstick sample (Vargas et al., 2022). This dual-detection capability is vital for distinguishing active infections (sensitivity 96.9% for high-titer cases) from treated historical exposure, enabling immediate “test-and-treat” clinical decisions (Vargas et al., 2022).

Nanotechnological Breakthroughs: A new frontier in biosensing involves silk cocoon membrane (SCM)-integrated ELISA (Duan, 2025). The high porosity and binding affinity of the SCM platform achieve 100% sensitivity, outperforming traditional microplate-based kits by enhancing antigen-antibody capture efficiency (Duan, 2025).

Digital Integration: To eliminate subjective visual interpretation errors, smartphone-based AI readers and cloud-based data synchronization are being integrated into POCT workflows (Vargas et al., 2022). These digital eHealth technologies facilitate real-time surveillance and ensure objective reporting in remote or resource-limited settings (Arrigo, 2023; Bristow and Klausner, 2025) (**Table 3**).

**Table 3:** Summary of current and emerging point-of-care (POC) and at-home syphilis testing platforms.

Platform name	Technology principle	Diagnostic target(s)	Regulatory status/approval	Key advantages	References
First to Know^®^ (FTK)	LFICT (Lateral Flow) - Fingerstick blood	TT (Treponemal) antibodies	FDA Approved (OTC)	First over-the-counter (OTC) self-test; high diagnostic accuracy for lay users.	(Clark et al., 2025)
DPP^®^ Syphilis Screen & Confirm	Dual Path Platform (DPP) technology	Combined NTT (non-treponemal) + TT antibodies	Clinical Application (e.g., CE Marked, FDA cleared contexts)	Simultaneously differentiates active infection from past/treated infection in one test.	(Vargas et al., 2022)
SCM-ELISA (Silk-based)	Silk cocoon membrane integration (Biosensor)	TT antibodies	Research/Prototype stage	Demonstrated superior sensitivity compared to standard commercial ELISA kits.	(Duan, 2025)
Abbott Determine™ HIV-1/2 Ag/Ab Combo	Rapid combo test card (Lateral Flow)	Multiplex HIV + Syphilis antibodies	WHO Prequalified	Multiplex prenatal screening; highly suitable for resource-limited settings.	(The ProSPeRo Network, 2022)
Smartphone-based Readers	AI image analysis/colorimetry of lateral flow results	Lateral flow test interpretation	Emerging Digital Trend/Research	Eliminates subjective interpretation errors; enables instant cloud data synchronization.	(Arrigo, 2023)

Ag/Ab, antigen/antibody; CLIA, chemiluminescence immunoassay; DPP, Dual Path Platform; ELISA, enzyme-linked immunosorbent assay; FDA, Food and Drug Administration; FTK, First to Know; HIV, human immunodeficiency virus; LFICT, lateral flow immunochromatographic test; NTT, nontreponemal test; OTC, over-the-counter; POCT, point-of-care testing; SCM, silk cocoon membrane; TT, treponemal test; WHO, World Health Organization.

## 6 Biomedical prevention and the AMR challenge

### 6.1 Biomedical prevention and the AMR challenge

A fundamental paradigm shift is underway, transitioning from reactive treatment to proactive biomedical prevention centered on doxycycline post-exposure prophylaxis (Gottlieb et al., 2024). Clinical trials have demonstrated that a single 200 mg dose of doxycycline within 72 hours of condomless sex can reduce syphilis incidence by over 70% in high-risk cohorts (Molina et al., 2018; Cornelisse et al., 2024). The breakthrough potential of Doxy-PEP lies in the broad-spectrum bacteriostatic action of tetracyclines, which inhibit protein synthesis by binding to the 30S ribosomal subunit of *T. pallidum*. However, its long-term sustainability is threatened by the potential for antimicrobial resistance (AMR) within the broader commensal microbiome, as selective pressure may enrich resistant determinants in species like *Staphylococcus aureus* or *Neisseria gonorrhoeae (*Bird et al., 2024). Future strategies must therefore balance these prophylactic benefits with rigorous longitudinal AMR monitoring and integrated antimicrobial stewardship (Gottlieb et al., 2024).

### 6.2 Vaccine development and the “stealth” pathogen challenge

The search for a protective vaccine remains the definitive goal for global disease eradication (Liu et al., 2024). Preclinical progress using reverse-vaccinology has identified promising outer membrane protein (OMP) candidates, such as Tp0954, an adhesin that has successfully induced sterile immunity in rabbit models (He et al., 2023). Despite these advances, human vaccine development is obstructed by the pathogen’s sophisticated immune evasion strategies, most notably the TprK antigenic variation system (Thean et al., 2022). TprK allows the spirochete to continually alter its surface epitopes, effectively creating a moving target for the host’s humoral response (Seña et al., 2024). To overcome these biological hurdles, future breakthroughs must align vaccine design with manufacturing scalability and global molecular epidemiology to ensure protection against diverse circulating strains (Waugh and Cameron, 2024).

### 6.3 Bridging the gap: AI-driven decision support and health equity

**Figure 3:**
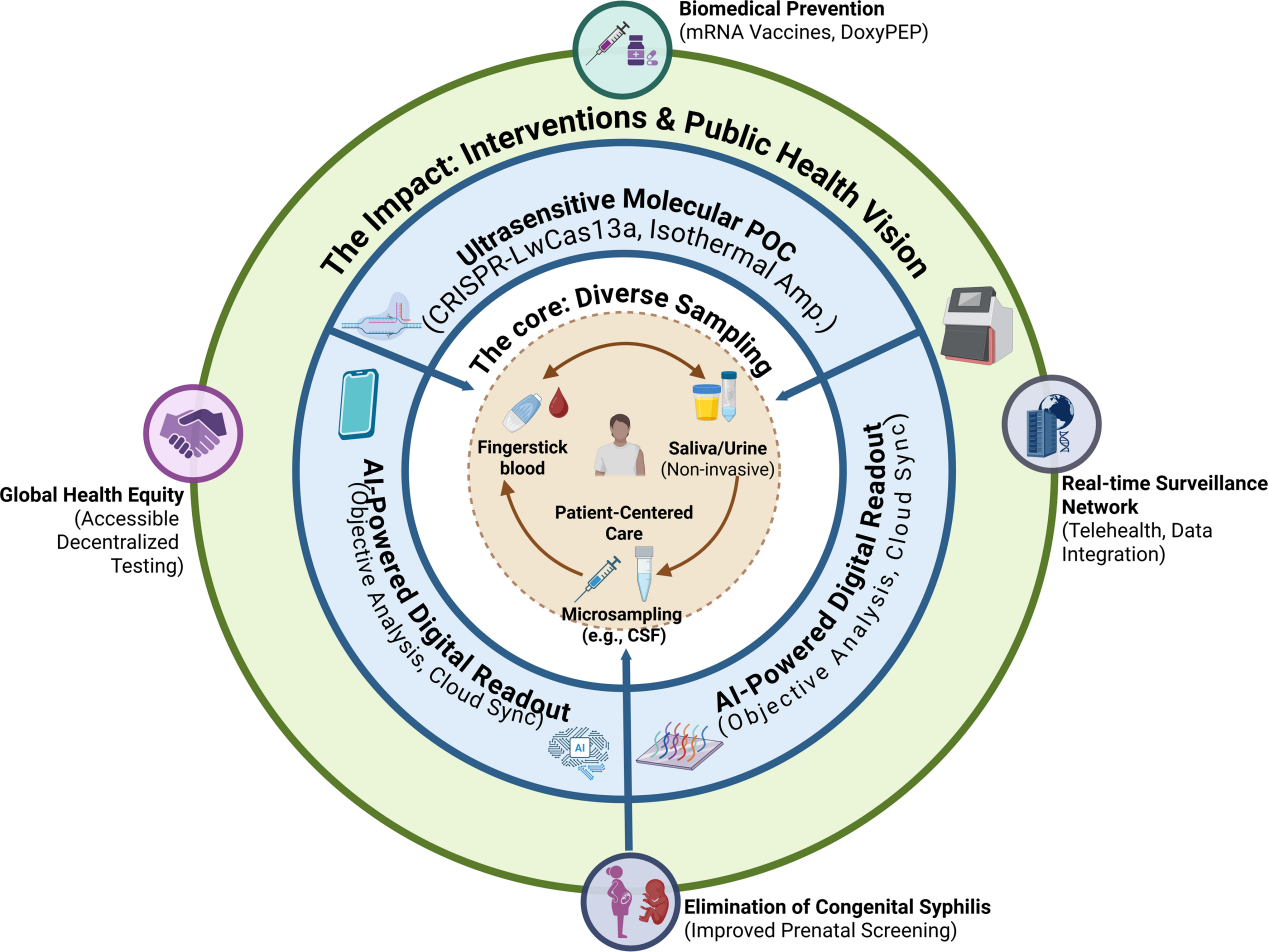
The future multimodal ecosystem and breakthrough pathways for syphilis diagnosis and management. This conceptual diagram illustrates a patient-centered diagnostic ecosystem driven by technological convergence. The Inner Core represents diverse, increasingly non-invasive sampling methods accessible at the point of care. The Middle “Innovation Engine” Ring highlights three clusters of breakthrough technologies: ultrasensitive molecular diagnostics (e.g., CRISPR), novel biosensors integrated with advanced materials (e.g., microfluidics), and AI-powered digital readers that ensure objective interpretation and connectivity. These innovations radiate outwards to empower The Outer Impact Ring, facilitating targeted biomedical interventions (Vaccines, DoxyPEP), enabling real-time digital surveillance networks, and ultimately aiming for the dual public health goals of eliminating congenital syphilis and achieving global health equity through decentralized access. AI, Antibody Index; AMR, Antimicrobial Resistance; BBB, Blood-Brain Barrier; CDC, Centers for Disease Control and Prevention; cfDNA, Cell-Free DNA; CMIA, Chemiluminescent Microparticle Immunoassay; CNS, Central Nervous System; CS, Congenital Syphilis; CSF, Cerebrospinal Fluid; CSF-VDRL, Venereal Disease Research Laboratory Test in Cerebrospinal Fluid; DALY, Disability-Adjusted Life-Years; DFM, Darkfield Microscopy; DoxyPEP, Doxycycline Post-Exposure Prophylaxis; EIA, Enzyme Immunoassay; ELISA, Enzyme-Linked Immunosorbent Assay; FDA, Food and Drug Administration; FTA-ABS, Fluorescent Treponemal Antibody Absorption; FTK, First to Know (Syphilis Test); HIV, Human Immunodeficiency Virus; IHC, Immunohistochemistry; LFICT, Lateral Flow Immunochromatographic Test; LISA, Luciferase Immunosorbent Assay; mNGS, Metagenomic Next-Generation Sequencing; MSM, Men who have Sex with Men; NAAT, Nucleic Acid Amplification Test; NGS, Next-Generation Sequencing; NS, Neurosyphilis; NTT, Nontreponemal Test; OMP, Outer Membrane Protein; OTC, Over-the-Counter; PCR, Polymerase Chain Reaction; POC, Point-of-Care; PrEP, Pre-Exposure Prophylaxis; QALY, Quality-Adjusted Life-Year; RDT, Rapid Diagnostic Test; RPR, Rapid Plasma Reagin; RSA, Reverse-Sequence Algorithm; S/Co, Signal-to-Cutoff (Ratio); SST, Syphilis Self-Testing; TA, Traditional Algorithm; TGW, Transgender Women; TMA, Transcription-Mediated Amplification; TP, Treponema pallidum; TPPA, Treponema pallidum Particle Agglutination; TRUST, Toluidine Red Unheated Serum Test; TT, Treponemal Test; VDRL, Venereal Disease Research Laboratory; WHO, World Health Organization.

Realizing the full potential of a multimodal ecosystem requires addressing the “diagnostic divide” through digital innovation and structural reforms (Caya et al., 2022). The integration of smartphone-based AI diagnostics and cloud-based synchronization offers a vision of real-time surveillance, but the complexity of interpreting discrepant serological results remains a bottleneck (Arrigo, 2023). A significant advancement in this domain is the development of the RSA-KG, a graph-based, RAG-enhanced AI knowledge graph designed to provide clinical decision support for complex diagnostic scenarios (He and Xiao, 2025). By leveraging large language models (LLMs) to synthesize evidence from disparate laboratory data, such tools can assist clinicians in low-resource settings to navigate the intricacies of the Reverse Sequence Algorithm (RSA) (He and Xiao, 2025). Ultimately, achieving global syphilis elimination requires a synergistic approach that combines rapid molecular diagnostics, proactive prevention, and digital infrastructure within a steadfast commitment to universal access and health equity (Paixao et al., 2023) (**Figure 3**).

## 7 Conclusion

The global resurgence of syphilis necessitates a paradigm shift from traditional diagnostic reliance toward an integrated, technology-driven framework. This review has demonstrated that the synergistic application of next-generation NAATs, such as CRISPR-based assays and TMA, offers a critical pathway to closing the serological window and enhancing detection in paucicellular stages. Furthermore, the optimization of serological algorithms through RSA and refined S/Co ratios provides a robust mechanism for reducing false positives and streamlining clinical workflows in high-throughput settings.

However, the transition toward a “multimodal diagnostic ecosystem” is not without challenges. As addressed in this review, the clinical implementation of these innovations remains hindered by significant economic barriers, regulatory gaps, and the operational complexity of decentralized testing. Moreover, the integration of biomedical prevention strategies like Doxy-PEP must be meticulously balanced against the emerging risk of antimicrobial resistance. Ultimately, achieving the goal of syphilis elimination requires not only technological breakthroughs but also a sustained commitment to health equity, ensuring that advanced diagnostics are accessible across diverse socio-economic landscapes (Peeling et al., 2023).
